# The high-affinity transporter BnPHT1;4 is involved in phosphorus acquisition and mobilization for facilitating seed germination and early seedling growth of Brassica napus

**DOI:** 10.1186/s12870-019-1765-3

**Published:** 2019-04-25

**Authors:** Ke-Lin Huang, Huan Wang, Ying-Li Wei, Han-Xin Jia, Lei Zha, Yong Zheng, Feng Ren, Xue-Bao Li

**Affiliations:** Hubei Key Laboratory of Genetic Regulation and Integrative Biology, School of Life Sciences, Central China Normal University, Wuhan, 430079 China

**Keywords:** *Brassica napus*, Phosphorus (P), Phosphate (pi), Abscisic acid (ABA), gibberellin (GA), Seed germination, Seedling growth and development

## Abstract

**Background:**

Seed germination and seedling establishment are two of the most critical phases in plant development. However, the molecular mechanisms underlying the effect of phosphorus on seed germination and post-germinated growth of oilseed rape are unclear so far. Here, we report the role of *BnPHT1;4* in seed germination and early seedling development of *Brassica napus*.

**Results:**

Our results show that *BnPHT1;4* is preferentially expressed in cotyledons of early developing seedlings. Overexpression of *BnPHT1;4* in oilseed rape promoted seed germination and seedling growth. Expression levels of the genes related to ABA and GA biosynthesis and signaling were significantly altered in *BnPHT1;4* transgenic seedlings. Consequently, active GA level was up-regulated, whereas ABA content was down-regulated in *BnPHT1;4* transgenic seedlings. Furthermore, exogenous GA could promote seed germination of wild type, while exogenous ABA could partially recover the advanced-germination phenotype of *BnPHT1;4* transgenic seeds. Total phosphorus content in cotyledons of the transgenic seedlings was decreased more rapidly than that in wild type when Pi was supplied or deficient, and Pi contents in shoots and roots of the *BnPHT1;4* transgenic plants were higher than those in wild type under high and low Pi conditions.

**Conclusions:**

Our data suggest that the high-affinity transporter BnPHT1;4 is involved in phosphorus acquisition and mobilization for facilitating seed germination and seedling growth of *Brassica napus* by modulating ABA and GA biosynthesis.

**Electronic supplementary material:**

The online version of this article (10.1186/s12870-019-1765-3) contains supplementary material, which is available to authorized users.

## Background

Seed germination is one of the most critical phases for the establishment of a plant [1]. A seed achieves germination when the appropriate nutrients conditions are present. Nutrient provision in seed germination and early seedling development only depends on storage products deposited in cotyledons at the end of seed (embryo) development before the root system have been established [2]. Previous studies revealed the influence of nutrients on seed germination. For instance, C and N control seed germination through interacting with phytohormones [3]. S-metabolism and ABA biosynthesis co-regulate seed germination of *Arabidopsis thaliana* [4]. K participates in maintaining turgor stability and osmoregulation of cells [5]. Zn, Fe, Cu, Ni, Mo, Mn, B and Cl take the structural roles in stabilizing proteins or act as catalytically active cofactors in enzymes [6]. However, little is known about the link of phosphorus nutrients and seed germination so far.

Phosphorus (P), the composition of membranelipids, energy carrier (ATP) and heredity (DNA and RNA), is an essential macro-element for plant growth and development [7–9]. Although a large amount of phosphorus exists in soil, the phosphate (Pi) readily available for plants is insufficient in soil [7]. Pi concentration within plant cells is usually above 10 mM (> 3 g/kg), but the concentration of Pi in most of the soil is typically lower than 10 μM [10]. Plants have evolved both high and low-affinity Pi transporters that mediate both P uptake and translocation in plants [11]. These Pi transporters belong to four subfamilies, PHT1 (phosphate transporter) to PHT4, and are plasma membrane proteins that are proton-coupled H_2_PO_4_^–^ symporters [12]. It was suggested that the high-affinity PHT1 family transporters are responsible for acquiring P from rhizospheres [13]. Unlike PHT1, proteins of the PHT2, PHT3 and PHT4 families are organelle Pi transporters and symport Pi into or out plastid (PHT2/4), mitochondrial (PHT3), or Golgi (PHT4) membranes [13]. Besides, some Pi transporters (such as AtPHT1;5) plays a critical role in mobilizing P between source and sink organs, and mobilizing the stored P out of older leaves crucial to maintain P homeostasis [14]. Expression analysis of the *PHT1* family members indicated that *AtPHT1;1*, *AtPHT1;3*, *AtPHT1;4*, *AtPHT1;5*, and *AtPHT1;6* are expressed in the hydathodes of cotyledons, suggesting a P transport system existed in cotyledons [15]. Histochemical assay revealed that strong GUS activity under the control of *AtPHT1;4* and *AtPHT1;5* promoters was detected throughout the cotyledons, suggesting *AtPHT1;4* and *AtPHT1;5* may be responsible for P mobilization in cotyledons [15]. Moreover, it has been reported that alteration of *OsPHT1;4* expression affected embryo development and had an effect on seed germination ability and subsequent seedling growth of rice [16].

Previous studies have extensively shown that phytohormones gibberellins (GA) and abscisic acid (ABA) are two of the primary endogenous factors regulating seed germination antagonistically. GA promotes the transition from seed dormancy to germination, whereas ABA inhibits this process [17–20]. In Arabidopsis, for instance, the impaired germination phenotype of seeds is observed in mutants (*ga1* and *ga2*) that are severely compromised in GA biosynthesis, while the loss-of-function of GA2OX2 (*ga2ox2* mutation) causes an increase in GA4 levels and promotes the germination of the mutant seeds [21, 22]. In contrast to GA mutants, seeds of the ABA-deficiency mutants (*nced3*, *nced5*, *nced6* and *aba2*) exhibit a faster-germination phenotype in Arabidopsis [23–25]. Furthermore, the ABA signaling mutants (*abi3*, *abi4* and *abi5*) of Arabidopsis showed the decreased seed dormancy and faster-germinating phenotypes [21, 26]. However, overexpression of the enhancer *ABI4* that is involved in ABA signal pathway inhibited seed germination of Arabidopsis [21]. Similarly, tobacco *ABA2* overexpression transgenic seeds exhibited the delayed-germination phenotype [27]. It has been indicated that the balance of ABA and GA determines the fate of a seed, dormancy or germination [19]. Additionally, N, P and S nutrients may be connected with hormone signaling during seed germination [3, 4].

In this study, we aim to reveal the possible phosphorus transport mechanism in cotyledons of *Brassica napus*. Our data indicated that expression level of *BnPHT1;4* is significantly higher than that of *BnPHT1;5* in cotyledons, so we focused on investigating the role of *BnPHT1;4* in early seedling development of *Brassica napus*. Although previous study reported the *BnPHT1;4* was involved in phosphorus uptake and roots architecture in the transgenic Arabidopsis plants [28], large amount of work is needed to dissect the biological roles of *BnPHT1;4* in *Brassica napus*. Our results revealed that overexpression of *BnPHT1;4* promotes seed germination and seedling growth of *Brassica napus* possibly by modulating ABA and GA biosynthesis. BnPHT1;4 is involved in phosphorus mobilization in cotyledons and phosphorus uptake from soil in roots during early seedling growth, indicating that BnPHT1;4 plays a critical role in phosphorus acquisition and mobilization for facilitating seed germination and early seedling growth of *Brassica napus*.

## Results

### *BnPHT1;4* is predominantly expressed in the cotyledons of *Brassica napus*

**Fig. 1:**
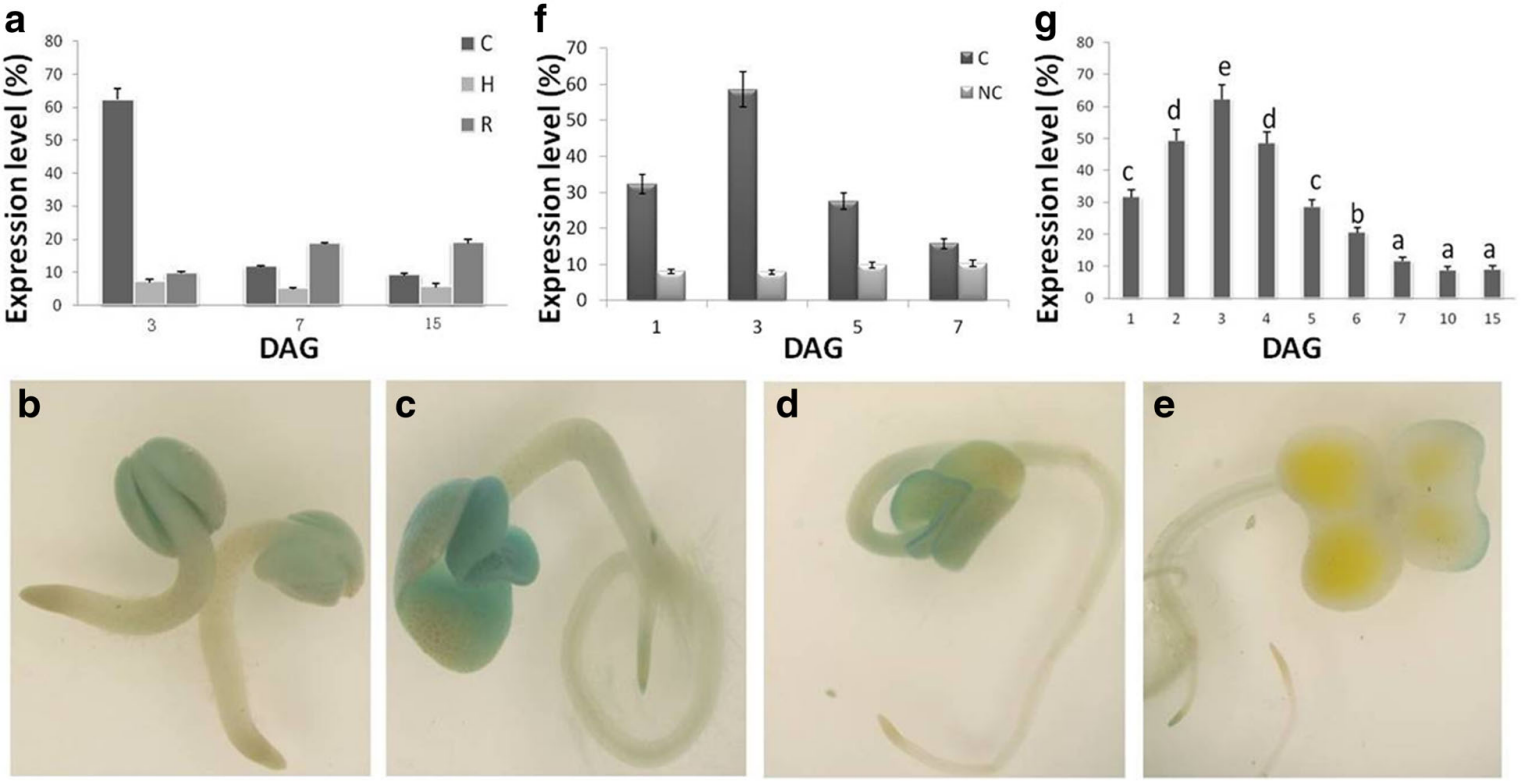
Expression profiling of *BnPHT1;4* in early developing seedlings of *Brassica napus*. (**a**) Quantitative RT-PCR analysis of *BnPHT1;4* expression in oilseed rape seedlings at 3–15 DAG. Total RNA was isolated from roots (R), hypocotyls (H), cotyledons (C) of seedlings cultured in normal conditions (1 mM Pi) at 3, 7 and 15 DAG for RT-PCR analysis, using *BnACT2* gene as standard control. The error bars indicate the standard errors (*n* = 3). (**b**-**e**). Histochemical assay of GUS activity in early developing seedlings. GUS staining observation of transgenic *B. napus* plants harboring the *BnPHT1;4* promoter: GUS fusion gene in 1 DAG (**b**), 3 DAG (**c**), 5 DAG (d) and 7 DAG (**e**) seedlings cultured on MS medium with 1 mM Pi, respectively. (**f**) Quantitative RT-PCR analysis of the *GUS* gene in cotyledons (C) and in roots plus leaves and hypocotyls (NC) of *BnPHT1;4 promoter: GUS* transgenic plants. Total RNAs were isolated from C and NC of 1 DAG, 3 DAG, 5 DAG and 7 DAG seedlings cultured on MS medium with 1 mM Pi, respectively, for RT-PCR analysis, using *BnACT2* gene as standard control. (**g**) Quantitative RT-PCR analysis of *BnPHT1;4* expression in cotyledons of oilseed rape seedlings at 1–15 DAG. Total RNAs was isolated from cotyledons of seedlings cultured in normal conditions (1 mM Pi) at 1–15 DAG for RT-PCR analysis, using *BnACT2* gene as standard control. The error bars indicate the standard errors (*n* = 9). Significance of difference was analyzed by Duncan’s test (*P* < 0.05). Different lowercase letters indicate statistically significant differences. DAG, day after germination

To reveal the possible phosphorus transport mechanism in cotyledons of *B. napus*, we first analyzed the expression levels of *BnPHT1;4* and *BnPHT1;5* in early developing seedlings of *B. napus*, and found that *BnPHT1;4* expression is significantly higher than that of *BnPHT1;5* during seed germination (Additional file 1 Figure S1). Then, we further analyzed expression of *BnPHT1;4* in tissues (such as cotyledons, hypocotyls and roots) at early seedling developmental stage. As shown in Fig. 1a, *BnPHT1;4* was predominantly expressed in cotyledons during early seedling development. To further elucidate the tissue-specific/preferential expression pattern of *BnPHT1;4*, we generated transgenic *B. napus* plants carrying a transcriptional fusion of the *BnPHT1;4* promoter and the reporter *GUS* gene. Histochemical assay revealed that GUS activity under the control of *BnPHT1;4* promoter was mainly detected in cotyledons, especially in very early seedling developmental stage after seed germination. As seedlings further developed, GUS activity was dramatically decreased in cotyledons, while no or very weak GUS staining was detected in roots and hypocotyls (Fig. 1b-e). Quantitative RT-PCR analysis also indicated that expression of the *GUS* reporter gene under the control of the *BnPHT1;4* promoter was mainly in cotyledons (Fig. 1f), suggesting that *BnPHT1;4* promoter is very active in cotyledons of the early developmental seedlings. Additionally, *BnPHT1;4* expression reaches to its peak value in cotyledons at three days after seed germination (DAG), and thereafter, gradually declined in cotyledons (Fig. 1g). Collectively, these results suggested that *BnPHT1;4* may play an important role in cotyledons during seed germination and early seedling development.

### *BnPHT1;4* is induced in cotyledons and roots under phophorus deficiency

**Fig. 2:**
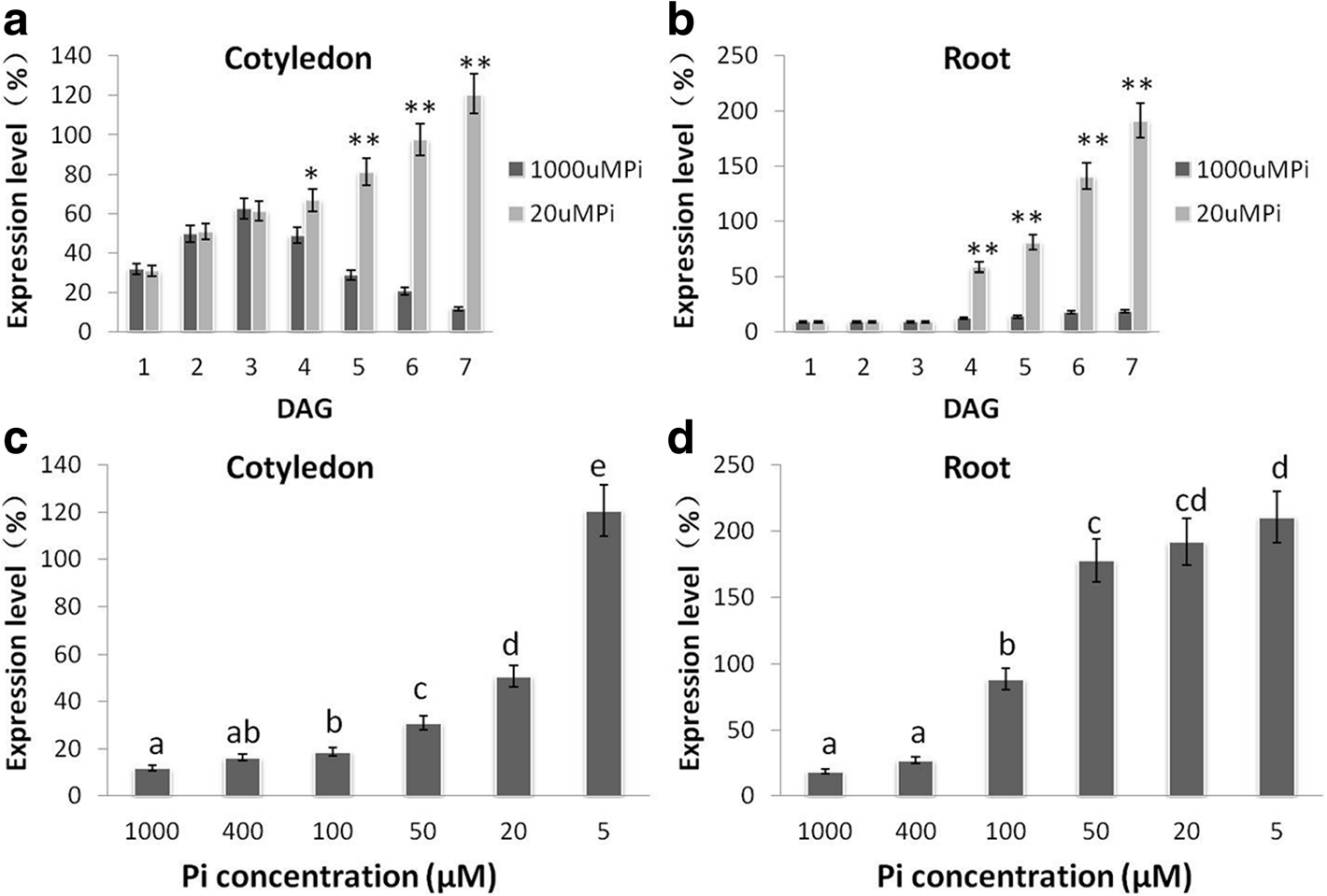
Expression profiling of *BnPHT1;4* in cotyledons and roots of *Brassica napus* under Pi starvation. (**a** and **b**) Quantitative RT-PCR analysis of *BnPHT1;4* expression in cotyledons (**a**) and roots (**b**) of oilseed rape seedlings at 1–7 DAG. Total RNA was isolated from cotyledons (**a**) and roots (**b**) of seedlings under 1000 or 20 μM Pi at 1–7 DAG for RT-PCR analysis, using *BnACT2* gene as standard control. (**c** and **d**) Quantitative RT-PCR analysis of *BnPHT1;4* expression in cotyledons (**c**) and roots (**d**) of oilseed rape seedlings under different Pi concentrations. The 7 DAG seedlings were transferred to 5, 20, 50, 100, 400 or 1000 μM Pi for 3 days, and then total RNA was isolated from cotyledons (**c**) and roots (**d**) for RT–PCR analysis, using *BnACT2* gene as standard control. KCl was used to replace KH_2_PO_4_ in the medium for the equivalent amount of potassium. The error bars indicate the standard errors. Significance of difference was analyzed by Duncan’s test (*p<0.05, **p<0.01, *n* = 3). Different lowercase letters indicate statistically significant differences. DAG, day after germination

*BnPHT1;4* is induced in *B. napus* under phosphorus (P) deficiency [28]. To further characterize *BnPht1;4* response to P concentrations during seed germination and seedling growth, we employed quantitative RT-PCR analysis to determine the expression of *BnPHT1;4* in roots and cotyledons at early developmental stages of seedlings under different Pi conditions. The expression of *BnPHT1;4* in both roots and cotyledons was not responsive to P deprivation before 3 DAG, but *BnPHT1;4* expression was dramatically induced under P-starvation condition after 3 DAG in both cotyledons and roots (Fig. 2a-b). Moreover, pre-germinated seedlings were grown for 15d on P-sufficient medium, and then transferred to medium with different Pi concentrations for 3d. *BnPHT1;4* expression in roots and cotyledons of these seedlings were analyzed by RT-PCR. As shown in Fig. 2c-d, transcriptional tendency of *BnPHT1;4* were similar to 5 DAG that *BnPHT1;4* is induced in cotyledons and roots under P deficiency. Moreover, pre-germinated seedlings grew on P-sufficient medium for 21 days, and then transferred to medium with 1 mM Pi or 20 μM Pi for 3 days. *BnPHT1;4* expression in roots and shoots of these seedlings were analyzed by quantitative RT-PCR. As shown in Additional file 1 Figure S2, expression level of *BnPHT1;4* was remarkably enhanced in both shoots and roots under P deficiency, relative to that under P-sufficient condition. These results indicate that *BnPHT1;4* is induced in cotyledons and roots of *B. napus* under P deprivation.

### Overexpression of *BnPHT1;4* in *B. napus* affects seed germination and seedling growth

**Fig. 3:**
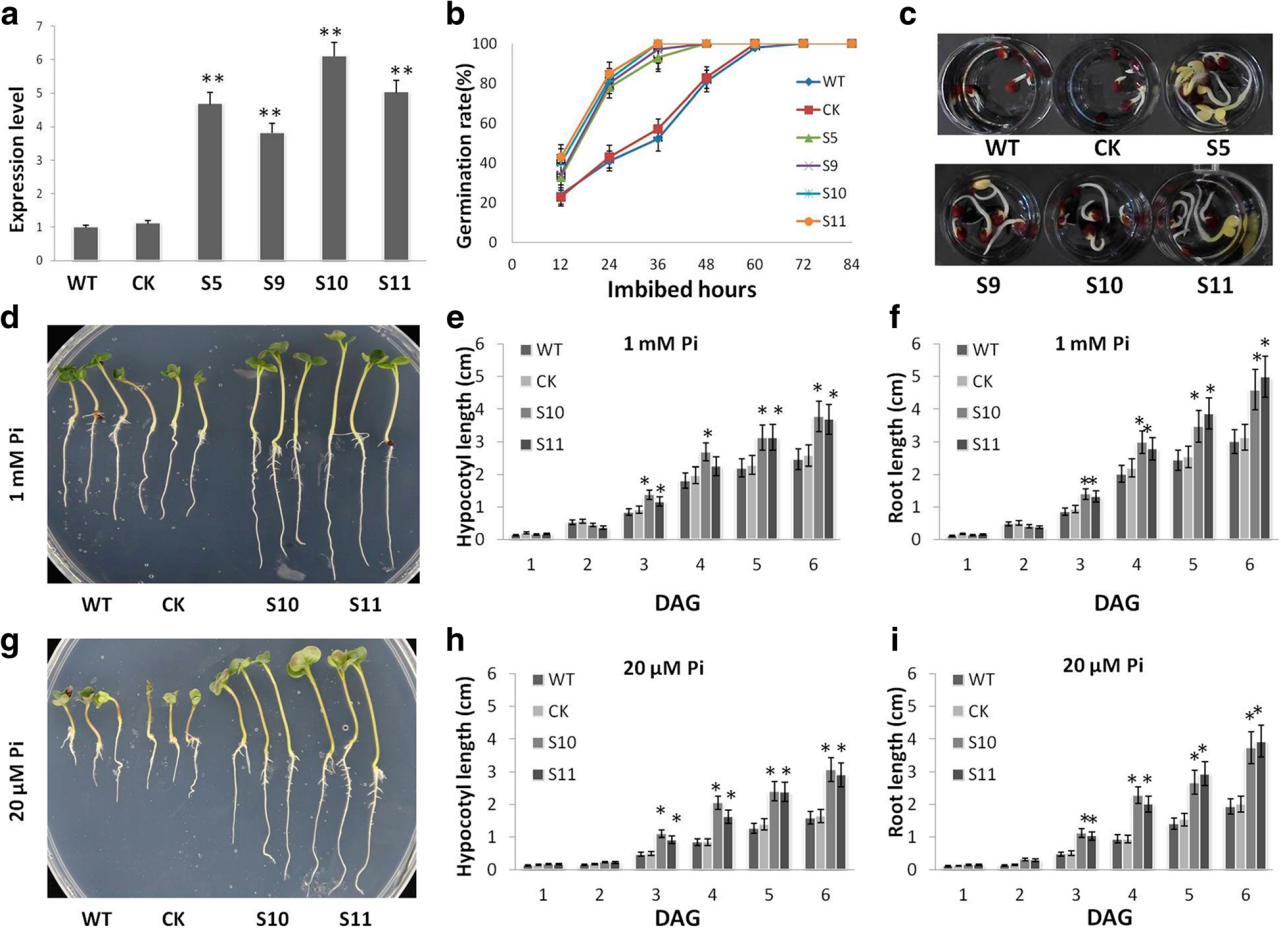
*BnPHT1;4* promotes seed germination and seedling growth of *Brassica napus*. (**a**) Detection of the transcript abundance of *BnPHT1;4* in the *BnPHT1;4* overexpression transgenic seedlings and controls (WT and CK) by quantitative RT-PCR analysis. Total RNA was isolated from roots and shoots of the 7 DAG seedlings. Expression levels of the *BnPHT1;4* in wild type are set to 1, and in the transgenic lines are shown in folds compared with that in wild type. The error bars indicate the standard errors. Significance of difference was analyzed by Duncan’s test (*p<0.05, **p<0.01, n = 3). (**b** and **c**) Overexpression of *BnPHT1;4* promotes seed germination. Seed germination of the *BnPHT1;4* overexpression transgenic lines and controls on 1/2 MS medium with 1000 μM Pi. Seeds were stored for 2 months after harvest and subjected to analysis. After seeds were sowed, quantitative analysis of germination rates at different time (hours of seed imbibition) are shown in b, and one representative image per genotype (1.5 days after sowing) is shown in c. (**d**-**i**) Overexpression of *BnPHT1;4* promotes early seedling growth. Seeds of the controls were sowed about 24 h earlier than those of *BnPHT1;4* transgenic lines, and then the seedlings with same length of radicle grew on 1/2 MS medium with 20 or 1000 μM Pi. KCl was used to replace KH_2_PO_4_ in the medium for the equivalent amount of potassium. One representative image per genotype (1.5 days after sowing) is shown in d (1000 μM Pi) and g (20 μM Pi). Quantitative analysis of hypocotyl length and root length in 1000 μM Pi are shown in e and f, while in 20 μM Pi are shown in h and i. Five experimental replications were performed and each replication contains at least l50 seedlings. The data are presented as means ± SD (*n* = 5). WT, wild type; CK, transgenic null line; S5, S9, S10 and S11, four independent *BnPHT1;4* overexpression transgenic lines. DAG, day after germination

To characterize the function of *BnPHT1;4* in P uptake and translocation in *B. napus*, we generated *BnPHT1;4* overexpression transgenic *B. napus* plants (OE*BnPHT1;4*) by *Agrobacterium tumefaciens*-mediated transformation. We selected four lines OE*BnPHT1;4*–5 (S5), OE*BnPHT1;4*–9 (S9), OE*BnPHT1;4*–10 (S10) and OE*BnPHT1;4*–11 (S11) for further characterization. The expression levels of *BnPHT1;4* were increased by 3- to 6-folds in OE*BnPHT1;4* lines compared with those in the wild type (WT) and transgenic null line (CK) (Fig. 3a).

We then examined seed germination of these four independent OE*BnPHT1;4* lines under P-sufficient (1 mM) conditions, using WT and CK as controls. As shown in Fig. 3b, OE*BnPHT1;4* seeds germinated at a faster rate than control seeds. We determined the resulting germination frequencies by counting the number of seeds with protruding radicles. The results indicated that seed germination of OE*BnPHT1;4* lines is faster than the controls (Fig. 3c). Similarly, the length of hypocotyls and primary roots of the transgenic seedlings was significantly increased, relative to that of the controls (Fig. 3d-i). However, overexpression of *BnPHT1;4* don’t affect the total P content and weight of rape seeds (Additional file 1 Figure S3 and Figure S4).

### Overexpression of *BnPHT1;4* enhances GA biosynthesis, but suppresses ABA biosynthesis

**Fig. 4:**
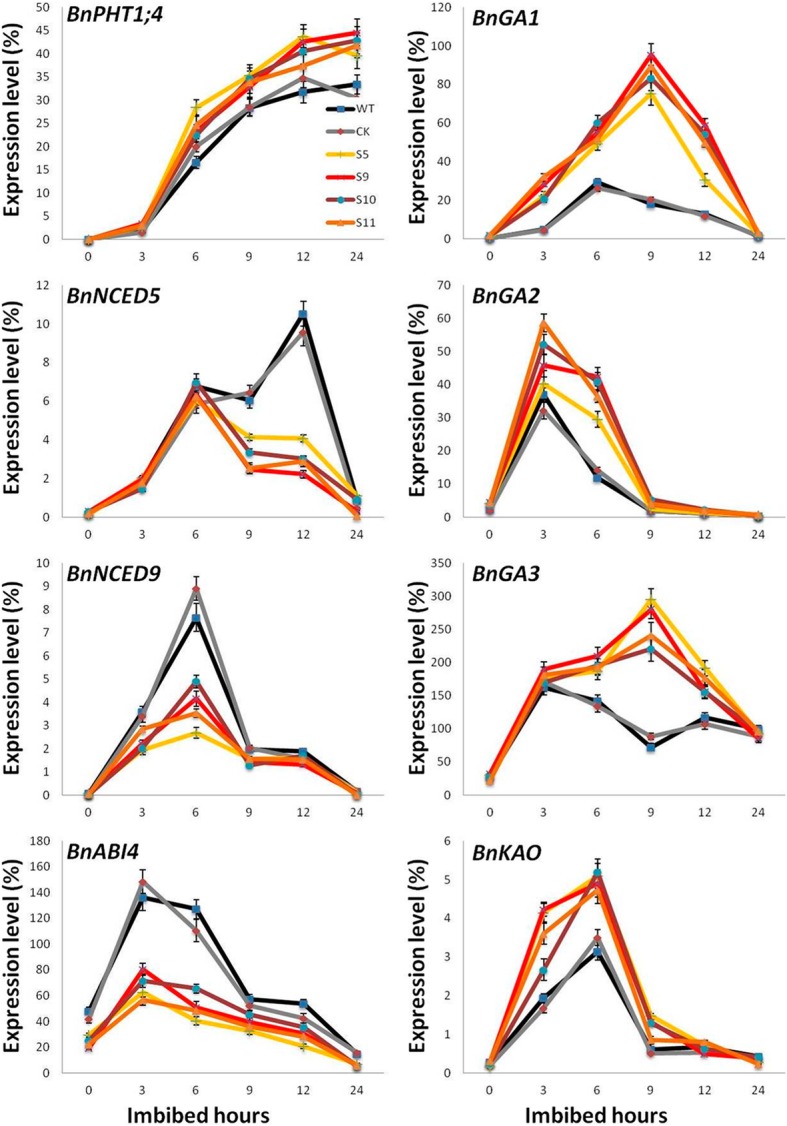
Quantitative RT-PCR analysis of gene expressions in dry and imbibed seeds of the *BnPHT1;4* overexpression transgenic *Brassica napus*. Gene expression of *BnPHT1;4*, GA-related genes (*BnGA1*, *BnGA2*, *BnGA3* and *BnKAO1*), ABA biosynthesis-related genes (*BnNCED5* and *BnNCED9*) and ABA signaling positive regulator gene (*BnABI4*) was determined in germinating seeds during the course of the imbibition process. Two-month stored seeds were used for seed germination, and total RNA was isolated from the dry and imbibed (germinating) seeds for RT-PCR analysis, using *BnACT2* gene as standard control. The data are presented as means ± SD (*n* = 3), and significance of difference was analyzed by Duncan’s test. WT, wild type; CK, transgenic null line; S5, S9, S10 and S11, four independent *BnPHT1;4* overexpression transgenic lines

**Fig. 5:**
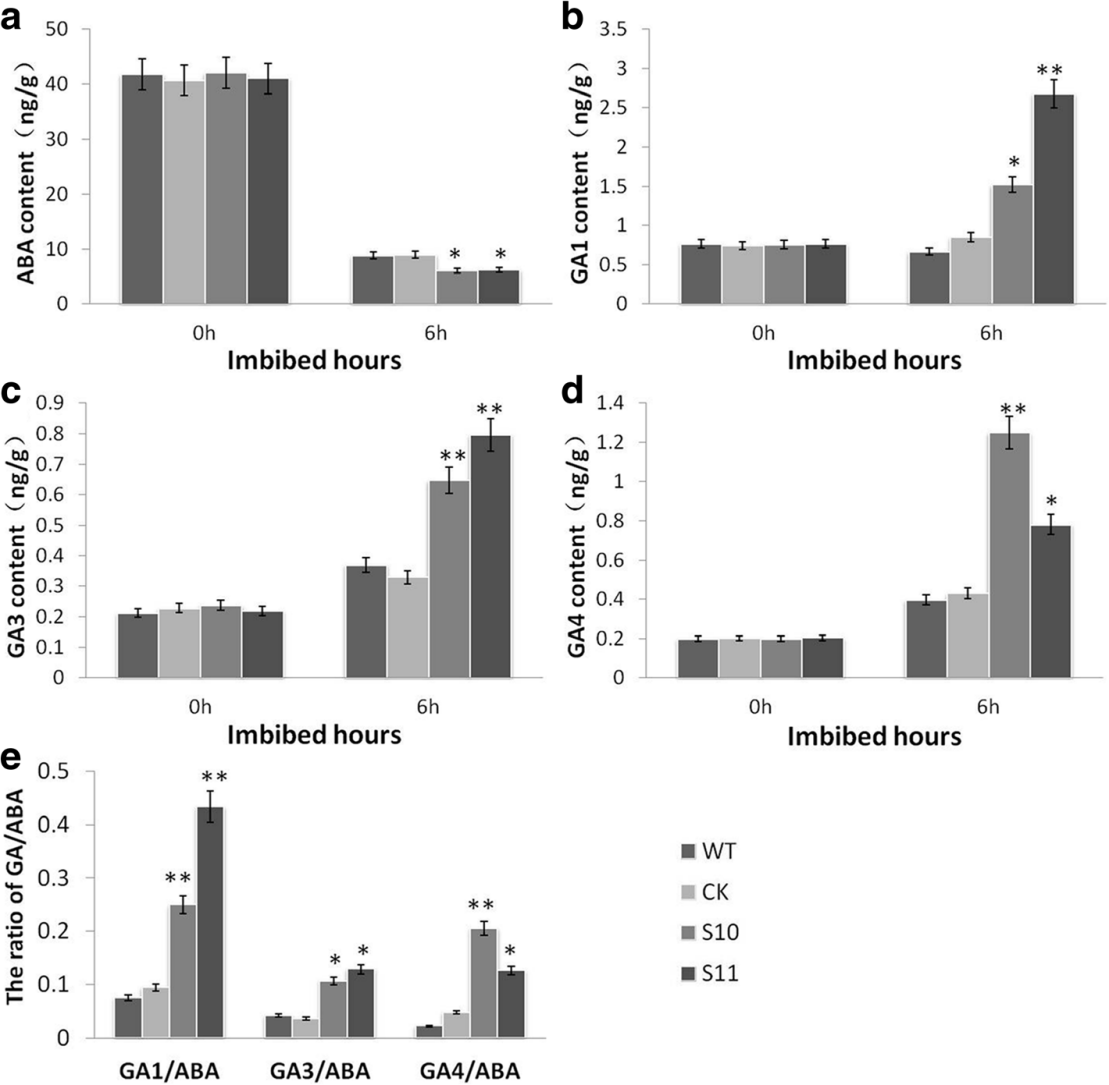
Assay of endogenous GA and ABA contents in *BnPHT1;4* overexpression transgenic seeds of *Brassica napus*. (**a**-**d**) Contents of ABA, GA1, GA3 and GA4 in dry and imbibed seeds of the transgenic lines (S10 and S11) and controls (WT and CK) (see Methods). (**e**) Ratios of GA1/ABA, GA3/ABA, and GA4/ABA in dry and imbibed seeds of the transgenic lines and controls. The error bars indicate the standard errors. Significance of difference was analyzed by Duncan’s test (*p<0.05, **p<0.01, *n* = 3). WT, wild type; CK, transgenic null line; S10 and S11, independent *BnPHT1;4* overexpression transgenic lines

Previous studies demonstrated that ABA and GA play key roles in seed germination [17, 18]. Therefore, we further investigated whether the effect of BnPHT1;4 on seed germination is related to GA and ABA pathways. We compared the dynamic changes in expression of genes involved in ABA and GA biosynthesis and signaling in the transgenic lines and wild type controls of *B. napus* during the imbibition process of seeds. Quantitative RT-PCR analysis showed that the expression levels of the ABA biosynthesis-related genes *BnNCED9* and *BnNCED5* were significantly decreased in the *BnPHT1;4* transgenic lines. Also, expression of *BnABI4* was declined in the transgenic lines, relative to wild type (Fig. 4). Subsequently, we examined the expression patterns of the key genes involved in GA biosynthesis and signaling pathway. The results showed that expression of *BnGA1*, *BnGA2*, *BnGA3* and *BnKAO* was enhanced in the *BnPHT1;4* transgenic lines at 3 to 12 h after imbibitions of seeds, compared with those in wild type (Fig. 4), suggesting *BnPHT1;4* probably positively regulates GA biosynthesis during seed germination of *B. napus*. Consequently, we determined the endogenous ABA and active GA levels during imbibition of seeds. The results indicated that overexpression of *BnPHT1;4* significantly decreased active ABA levels in the imbibed transgenic seeds (Fig. 5a). In contrast, the GA1, GA3, and GA4 concentrations in the imbibed transgenic seeds were remarkably increased during imbibition, compared with those in the controls (Fig. 5b-d), and ratios of GA1/ABA, GA3/ABA and GA4/ABA were up-regulated (Fig. 5e). Altogether, the results suggest that *BnPHT1;4* may negatively modulate ABA biosynthesis but positively regulates GA biosynthesis in *B. napus*.

**Fig. 6:**
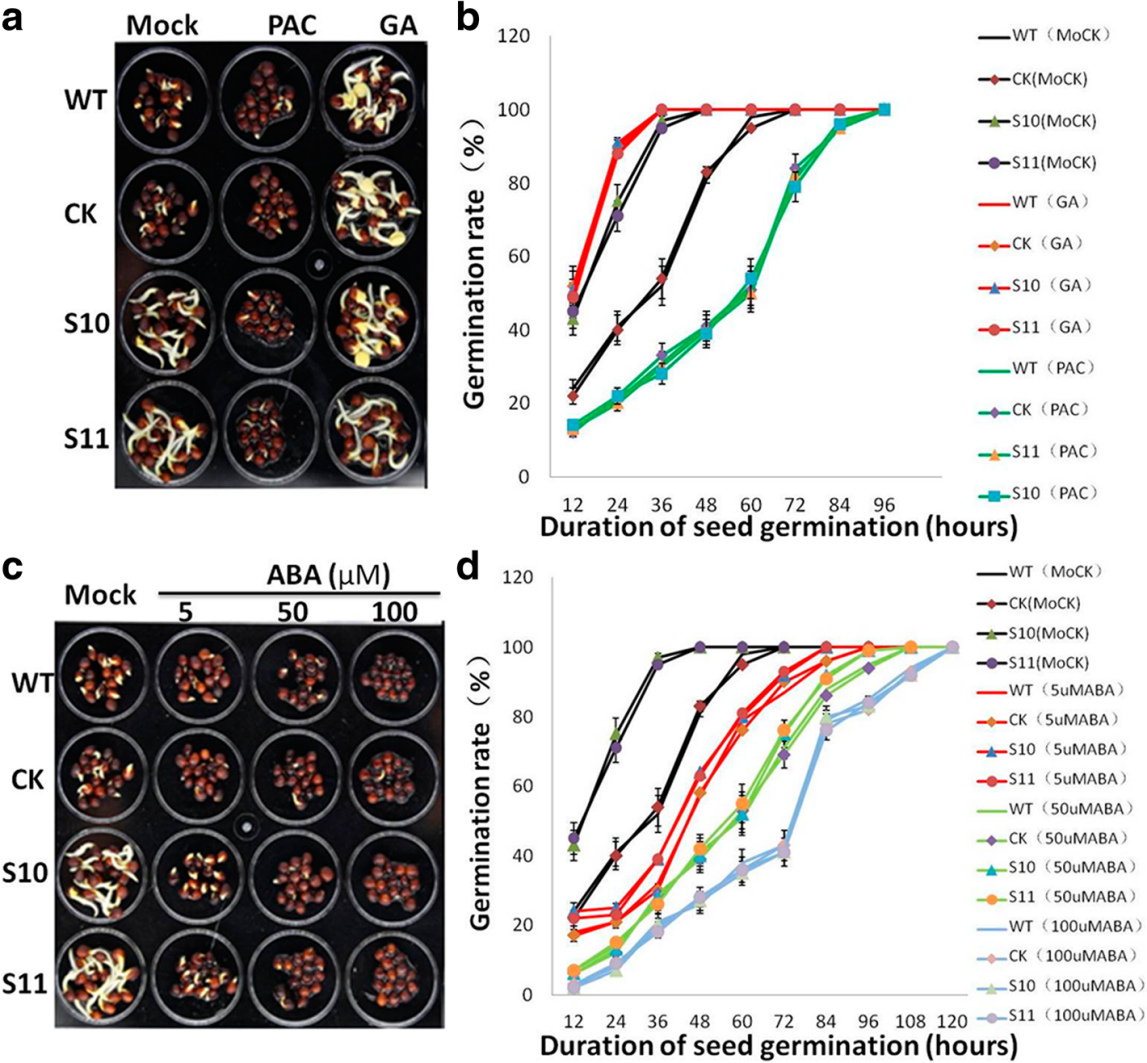
Effects of GA and ABA on recovering the advanced-germination phenotype of the *BnPHT1;4* overexpression transgenic *Brassica napus* seeds. (**a** and **b**) effects of GA and PAC (paclobutrazol, a GA biosynthesis inhibitor) on seed germination. Seeds were treated with 0.1 mM GA or 10 mM PAC. One representative image per genotype (1.5 days after sowing) is shown in a. Quantitative analysis of germination rates are shown in b. The *BnPHT1;4* transgenic seeds (S10 and S11) germinated more rapidly than those of the controls (WT and CK) under normal conditions (Mock). PAC delayed germination of the *BnPHT1;4* transgenic seeds, while GA promoted seed germination of the controls. (**c** and **d**) effects of ABA on seed germination. Seeds were treated with 5, 50 and 100 μM ABA. One representative image per genotype (1.5 days after sowing) is shown in c. Quantitative analysis of germination rates are shown in d. ABA partially recovered the advanced-germination phenotype of the *BnPHT1;4* transgenic seeds. The data are presented as means ± SD (n = 5). WT, wild type; CK, transgenic null line; S10 and S11, independent *BnPHT1;4* overexpression transgenic lines

To further confirm the role of *BnPHT1;4* in GA and ABA biosynthesis during seed germination, we tested the responsiveness of rape seeds to ABA, GA and paclobutrazol (PAC, a GA biosynthesis inhibitor) during seed imbibition. We supposed that the advanced-germination phenotype of the *BnPHT1;4* overexpression transgenic seeds is caused by the enhanced GA biosynthesis. The experimental results indicated that PAC could partially delay the advanced-germination phenotype of the *BnPHT1;4* transgenic seeds. The effects on post-germination growth parameters, such as radical length, were also similar to the germination results. Furthermore, exogenous GA could narrow the gap of seed germination velocity between the transgenic lines and controls (Fig. 6a-b and Additional file 2 Dataset S4). On the other hand, exogenous ABA inhibited seed germination of both transgenic lines and wild type controls, and reduced the index of germination velocity of the transgenic seeds and controls. Moreover, exogenous ABA could partially weaken the effect caused by overexpression of *BnPHT1;4*. This trend was more pronounced at higher ABA concentrations, especially when the ABA concentrations reach to 100 μM (Fig. 6c-d and Additional file 2 Dataset S3). Taken together, these results revealed that overexpression of *BnPHT1;4* promotes seed germination process of *B. napus* by suppressing ABA biosynthesis while promoting GA biosynthesis.

### BnPHT1;4 is involved in phosphorus mobilization in seedlings and phosphorus uptake from soil

**Fig. 7:**
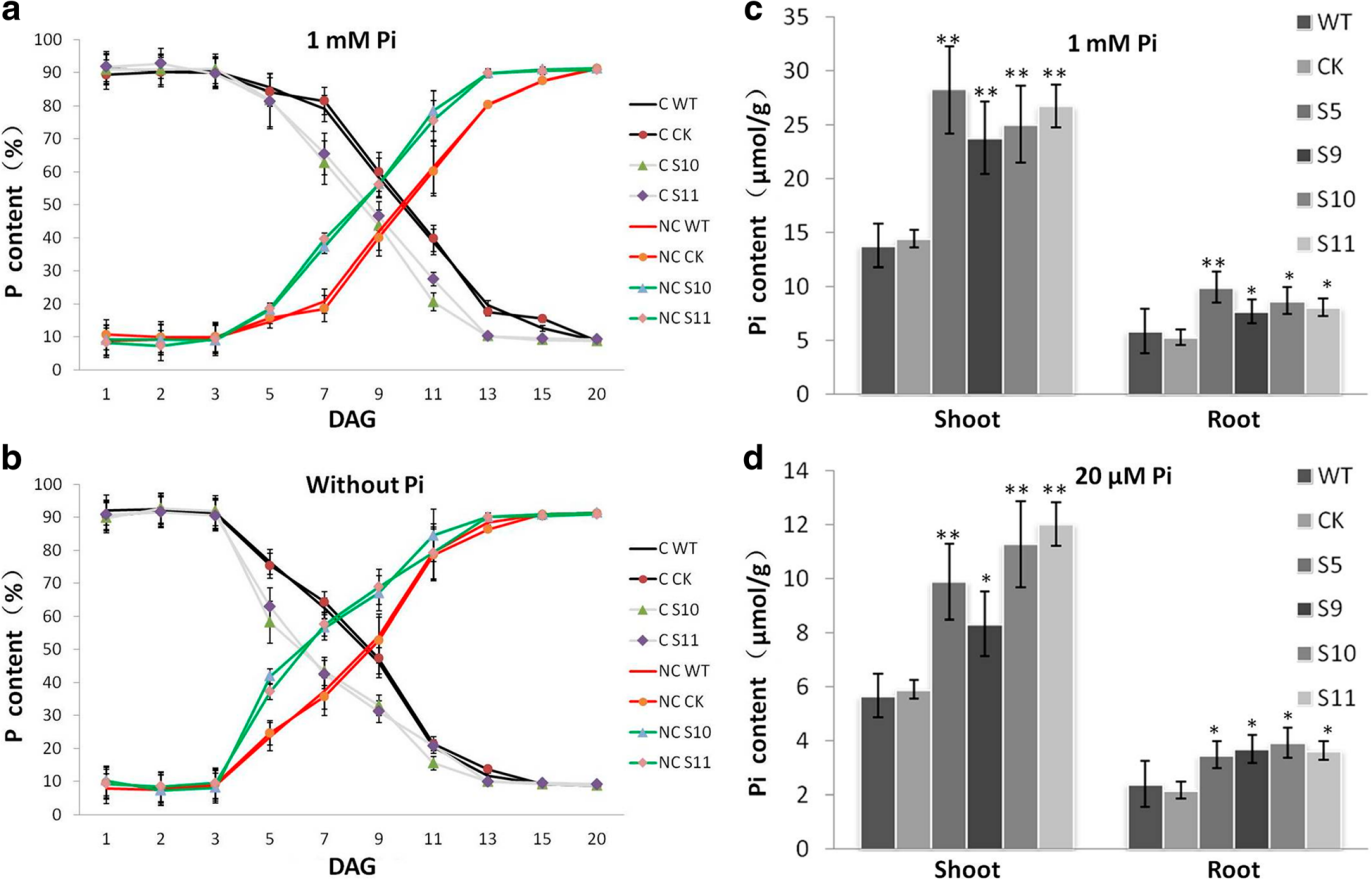
*BnPHT1;4* is involved in phosphorus mobilization in cotyledons and phosphorus uptake in roots of *Brassica napus*. (**a** and **b**) Dynamics of the relative pool sizes of the total phosphorus (P) in oilseed rape seedlings of the *BnPHT1;4* overexpression transgenic lines and controls (WT and CK). Seedlings grew on 1/2 MS medium with 1 mM Pi (**a**) or without Pi (**b**). P content was determined separately in cotyledons (**c**) and in roots plus leaves and hypocotyls (NC), and depicted as percentage of total P content in the whole seedling. The data are presented as means ± SD (*n* = 3). (**c** and **d**) Phosphate (Pi) contents in shoots and roots of seven-day-old seedlings of the *BnPHT1;4* overexpression transgenic lines and controls grown under 1 mM Pi (**c**) and 20 μM Pi (d) conditions. KCl was used to replace KH_2_PO_4_ in the medium for the equivalent amount of potassium. The error bars indicate the standard errors. Significance of difference was analyzed by Duncan’s test (*p<0.05, **p<0.01, *n* = 9). WT, wild type; CK, transgenic null line; S5, S9, S10 and S11, independent *BnPHT1;4* overexpression transgenic lines. DAG, day after germination

As *BnPHT1;4* is abundantly expressed in cotyledons, and overexpression of *BnPHT1;4* in *B. napus* affects seed germination, we deduce that *BnPHT1;4* may be involved in P mobilization in cotyledons that supply P for the formation of new organs. So, we analyzed total phosphorus contents in cotyledons and non-cotyledons (including roots, hypocotyls and new leaves) at the stages of seed germination and early seedling growth. As shown in Fig. 7a and Additional file 2 Dataset S1, total phosphorus was constantly decreased in cotyledons of both OE*BnPHT1;4* lines and controls. Moreover, total phosphorus content in cotyledons of transgenic plants was declined more rapidly than that in the wild type and CK controls. To further demonstrate the role of *BnPHT1;4* in phosphorus mobilization in cotyledons, we analyzed total phosphorus dynamics during seed germination and early seedling growth without exogenous Pi supply (Fig. 7b and Additional file 2 Dataset S2). In the absence of exogenous phosphorus, total phosphorus content in cotyledons was decreased more rapidly in both transgenic plants and controls, suggesting that there is a mechanism of phosphorus transport in cotyledons response to phosphorus change. Furthermore, total phosphorus content in the transgenic plants was decreased faster than that in the controls when no exogenous Pi was supplied in solution, indicating *BnPHT1;4* may play an important role in phosphorus mobilization in early developing seedlings. Moreover, Pi contents in shoots and roots of *BnPHT1;4* transgenic plants were remarkably higher than those in the controls under high and low Pi conditions, indicating that the high-affinity transporter BnPHT1;4 in roots may be responsible for Pi uptake from soil (Fig. 7c-d).

Additionally, rape seeds germinated on 1/2 MS medium with different Pi content. As shown in Additional file 1 Figure S5a, exogenous Pi did not affect the seed germination process of *B. napus*. There was no significant difference in germination rates of rape seeds among the different Pi conditions. On the other hand, post-germinated growth rate of seedlings in high Pi content was faster than that in Low Pi content. P deficiency significantly reduced the hypocotyl and root length of seedlings (Additional file 1 Figure S5b-d). The above results indicated that exogenous phosphorus don’t affect seed germination but affect seedling growth of *B. napus*.

### Expression of *BnPAP2* and *BnPAP15* are altered in *BnPHT1;4* transgenic plants

**Fig. 8:**
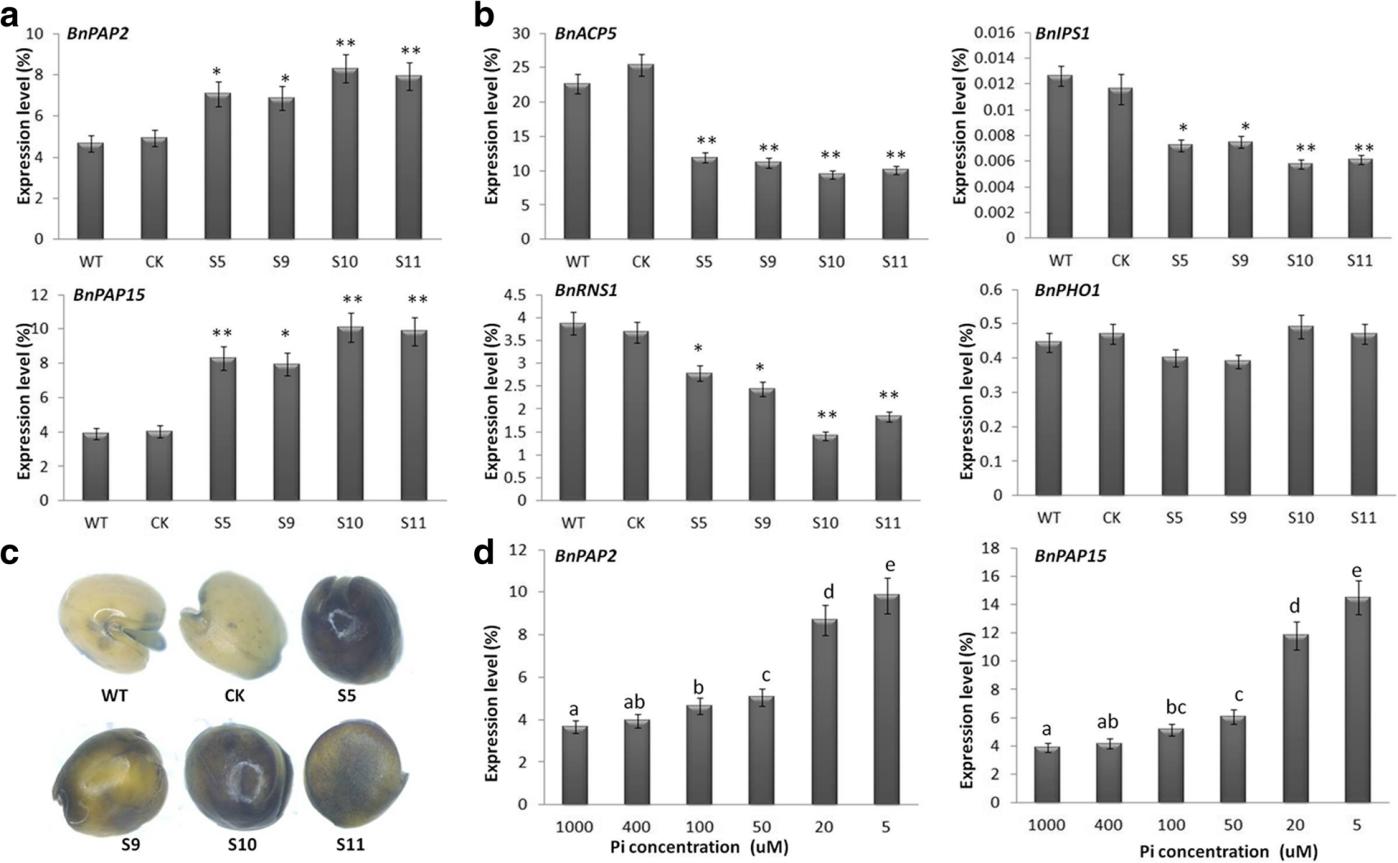
Quantitative RT-PCR analysis of expression of *BnPAP2* and *BnPAP15* in *BnPHT1;4* overexpression transgenic *Brassica napus*. (**a** and **b**) Expression of *BnPAP2* and *BnPAP15* (**a**) and *BnACP5*, *BnIPS1*, *BnRNS1* and *BnPHO1* (**b**) in 9-h-imbibed seeds of the *BnPHT1;4* overexpression transgenic lines and controls (WT and CK) on 1/2 MS medium with 1000 μM Pi. (**c**) BCIP staining assay of phosphatase activity in 9-h-imbibed seeds of the *BnPHT1;4* overexpression transgenic lines and controls (WT and CK) on 1/2 MS medium with 1000 μM Pi. (**d)** Expression of *BnPAP2* and *BnPAP15* in cotyledons of oilseed rape seedlings with different Pi treatments. The seven-day-old WT seedlings were transferred into liquid medium with 5, 20, 50, 100, 400, 1000 and 2000 μM Pi for 2 days, and then total RNA was isolated from cotyledons for RT-PCR analysis, using *BnACT2* gene as standard control. KCl was used to replace KH_2_PO_4_ in the medium for the equivalent amount of potassium. The error bars indicate the standard errors. Significance of difference was analyzed by Duncan’s test (*p<0.05, **p<0.01, *n* = 3) in a and b, and different lowercase letters indicate statistically significant differences in d (*P* < 0.05, *n* = 3)

Phosphorus stored in plant seeds is mainly in the form of phytic acid (myo-inositol hexakisphosphate). During seed germination, phytic acid is degraded by phytase to yield inositol and free phosphate provisions in early seedling development [29]. Therefore, there may be a link between Pi transporter and phytases. As shown in Fig. 8a-b, both *BnPAP2* and *BnPAP15* were up-regulated in the *BnPHT1;4* overexpression seedlings of *B. napus*. The 5-bromo-4-chloro-3-indolyl phosphate (BCIP) staining also showed that the phosphatase activity were significantly increased in the *BnPHT1;4* transgenic lines, compared with that in the controls (Fig. 8c). However, *BnIPS1*, *BnACP5* and *BnRNS1* were down-regulated in the *BnPHT1;4* overexpression seedlings of *B. napus* (Fig. 8b). Moreover, pre-germinated seedlings grew on Pi-sufficient (1 mM) medium for 7 days and then transferred to MS medium with different Pi contents for 2 days. Cotyledons of these seedlings were selected to investigate the expression of *BnPAP2* and *BnPAP15*. As shown in Fig. 8d and Additional file 1 Figure S6, expression of *BnPAP2* and *BnPAP15* was induced in cotyledons of both transgenic lines and controls under Pi deficiency conditions. These results suggested that overexpression of *BnPHT1;4* promotes P release from cotyledons of *B. napus*.

## Discussion

It is well-known that the major phosphorus nutrient is restored in cotyledons of rape seeds [2]. Phosphorus nutrient provision in early developing seedlings depends on reallocated nutrient reserves before the root system have been established [30]. So, a P translocation system may exist in plants for transporting phosphorus from cotyledons to roots/hypocotyls during seed germination and early seedling development. So far, multiple P transporters involved in phosphorus uptake from soil and phosphorus translocation within the plant have been identified [31]. For example, AtPHT1 is involved in phosphorus uptake from soil, AtPHO1 transporters are responsible for loading Pi into xylem of roots and transferring phosphorus from the chalazal seed coat to the embryo in developing seeds, and AtPHT1;5 takes part in mobilizing stored phosphorus out of older leaves [32–34]. However, the P transporter responsible for phosphorus translocation out of cotyledons during seed germination is not clear yet, especially in rape seeds. In this study, we found that *BnPHT1;4* is strongly expressed in cotyledons during rape seed germination and early seedling growth in normal Pi conditions, suggesting that *BnPHT1;4* functions in cotyledons for phosphorus transport. The transcripts of *BnPHT1;4* are gradually increased in cotyledons after imbibition of the dry seeds and reach to its peak value before the cotyledons turn green, during which root system is not established for nutrient uptake.

In higher plants, the high-affinity P transport system is regulated by the status of phosphorus, different from the constitutively expressed low-affinity P transport system [35]. Previous studies revealed that Arabidopsis *PHT1;1* and *PHT1;4* are induced in roots under phosphorus deficiency [36], and *BnPHT1;4* is highly expressed in P-starved roots [28]. Our data indicated that expression level of *BnPHT1;4* is not changed in P deficient conditions when the cotyledons remain yellow, suggested that phosphorus nutrient provision in the early developing seedlings mainly depends on reallocated phosphorus reserves. On the other hand, *BnPHT1;4* expression was significantly increased under P deficiency in both cotyledons and roots after the cotyledons turned green (Fig. 2). Induction of *BnPHT1;4* expression by P deficiency in cotyledons and roots revealed that BnPHT1;4 may play an important role in both cotyledons and roots to cope with phosphorus limitations in the environment for seedling growth. Additionally, these results also suggested that the time of cotyledons turned green may be the critical point that phosphorus nutrient provision in germinated seeds and early developing seedlings mainly depends on reallocated phosphorus stored in cotyledons or acquire phosphorus from the rhizosphere.

The data of a time-course analysis showed that the total phosphorus content was constantly decreased in cotyledons of both *BnPHT1;4* transgenic plants and wild type controls (Fig. 7). This is consistent with the results described by Eggert and colleagues [2]. The fact that phosphorus was consumed faster in cotyledons of oilseed rape seedlings without exogenous phosphorus supply indicated there is a P translocation system in cotyledons. Moreover, the total phosphorus content in cotyledons of *BnPHT1;4* transgenic plants was decreased more rapidly than that in the controls when P nutrition was supplied or deficient, indicating that BnPHT1;4 may be responsible for phosphorus translocation in cotyledons to provide phosphorus nutrient for early seedling development of *B. napus*. On the other hand, Pi contents in shoots and roots of the *BnPHT1;4* transgenic seedlings were higher than that of wild type under high and low Pi conditions. And the hypocotyls and primary roots of the transgenic seedlings are significantly longer than those of the wild type controls. These results suggested that high-affinity transporter BnPHT1;4 may be responsible for phosphorus uptake from soil for seedling development. Also, our data suggested that BnPHT1;4 may perform dual functions in oilseed rape seedlings. It is responsible for phosphorus translocation in cotyledons before the cotyledons turn green and the root system is not established, and is responsible for phosphorus mobilization in cotyledons and phosphorus uptake in roots after the root system is established for nutrient uptake from soil.

It has been reported that seed germination is influenced by some endogenous factors (such as seed genotype, dormancy and hormone status) as well as some environmental conditions (such as moisture, oxygen and temperature) [37]. During seed germination, storage nutrients, such as lipids, proteins and others, are catabolized for maintaining early seedling development. Seed size and weight are correlated positively to seed germination and seedling establishment [38]. Larger canola seeds have higher levels of the measured mineral elements, in particular with those elements (such as K, P, S, Mg and Mn enriched in cotyledons, than those smaller seeds. A significant reduction in phosphorus content was found in those seeds that required more time for germination and seedling growth [2]. However, molecular mechanisms underlying the effect of phosphorus on seed germination and post-germinated growth of oilseed rape are still largely unclear in detail. In this study, our data revealed *BnPHT1;4* may be responsible for phosphorus translocation in cotyledons during seed germination. Overexpression of *BnPHT1;4* increased the phosphorus flow from cotyledons (source) to the most rapidly growing tissues and organs (sinks), thereby accelerating seed germination of *B. napus*.

Seed germination depends on the right phytohormonal response in the seed. Previous studies indicated that GA and ABA are two primary endogenous factors regulating seed germination [17–20]. In this study, gene expression analysis and phytohormones measurement demonstrated that BnPHT1;4 positively regulates GA biosynthesis, but negatively modulates ABA biosynthesis in early developing seedlings, leading to an increase in the GA/ABA ratios (including GA1/ABA, GA3/ABA, and GA4/ABA). Moreover, exogenous GA could narrow the gap of germination velocity between the transgenic seeds and wild type controls, whereas the decreased endogenous GA level by PAC (a GA biosynthesis inhibitor) could partially recover the advanced-germination phenotype of the *BnPHT1;4* transgenic seeds. Also, ABA could partially weaken this advanced-germination phenotype caused by overexpressing *BnPHT1;4*. Altogether, our data suggested that BnPHT1;4 may participate in regulating GA/ABA ratios to promote seed germination of *B. napus*.

## Conclusion

In brief, *BnPHT1;4* plays a critical role in phosphorus acquisition and mobilization to promote seed germination and early seedling growth of *Brassica napus* possibly by modulating ABA and GA biosynthesis. Our data presented in this study give a new insight into understanding the role of P transporter in *B. napus* and the effect of phosphorus on seed germination and post-germinated growth of oilseed rape. Also, this study provides a clue for further investigating the relationship of phosphorus and phytohormones ABA/GA balance during seed germination of *B. napus*.

## Methods

### Plant materials

Plants of oilseed rape (*Brassica napus* cv. *Westar*) and all the related transgenic lines grew in the experimental field in campus of Central China Normal University, Wuhan, China.

For seed germination phenotypic analysis, 100 seeds were cultured on a modified 1/2MS medium (containing 10.305 mM NH_4_NO_3_, 0.75 mM MgSO_4_, 9.395 mM KNO_3_, 1.495 mM CaCl_2_, 3% (*w*/*v*) sucrose, and 0.8% (w/v) agarose, PH 5.8) supplemented with different concentration of Pi in greenhouse (24 °C, 16 h light/8 h dark). Additionally, 1.25 mM KCl was used to replace KH_2_PO_4_ in the medium for the equivalent amount of potassium. Then, 10, 50 and 100 μM abscisic acid (ABA), 100 μM gibberellins (GA) or 10 mM paclobutrazol (PAC) was added in the medium according to the experiment requirements. Seed germination was considered when the radicle broke through the seed coat. For each germination test, five experimental replications were performed. Moreover, cotyledons, hypocotyls and roots at different developmental stages were harvested and used for length measurement, RNA isolation and P content assay. ABA, GA, and PAC (product number 33371) were ordered from Sigma–Aldrich Company Ltd., USA.

The oilseed rape seeds used in this study were provided by our lab.

### Vector construction and oilseed rape transformation

The vector construction of pBI121-BnPHT1;4 and BnPHT1;4p:GUS is same with our previous report [28]. All constructs were transferred into *Agrobacterium tumefaciens* strain GV3101 that was then used for plant transformation. Transformation of oilseed rape (*B. napus*) was carried out as described earlier [39]. The selective MS medium containing 50 mg/L kanamycin was used for screening the transformed seeds of *B. napus*, and the homozygous progeny plants (T3 and T4 generations) were used in the further experiments.

### Histochemical assay of GUS activity

Histochemical assay of GUS activity was performed according to a modified protocol [40]. Imbibition seeds of *BnPHT1;4p:GUS* transgenic oilseed rape were collected at different time points after sowing under different Pi conditions. The samples were incubated in GUS reaction buffer containing 5-bromo-4-chloro-3-indolyl-β-dglucuronic acid (X-Gluc) at 37 °C for 12–48 h. Then, 70% ethanol was used for clearing chlorophyll in the green tissues of seedlings. Images were documented using a Leica MZ16f stereomicroscope (Leica, Germany).

### Quantitative RT-PCR analysis

Cotyledons and roots of imbibition seeds were collected at different time points after sowing, and total RNA was extracted using RNeasy Mini kit (Qiagen, German). Expression of genes was analyzed by real-time RT-PCR using the fluorescent intercalating dye SYBR Green in a detection system. A two-step RT-PCR procedure was performed according to a method described earlier [41]. All data were normalized against the expression level of the *BnActin2* (*BnACT2*), and three biological replicates were performed for each experiment. The gene-specific primers used in the RT-PCR analysis are shown in Additional file 1 Table S1.

### Quantification of endogenous abscisic acid (ABA) and gibberellins (GA)

The dry seeds and imbibed seeds were frozen in liquid nitrogen, ground to fine powder, and extracted with 10 mL extraction buffer (ethyl alcohol/hydrochloric acid). 8 ng D6-ABA and GA d2 isotope standards (OIChemIm Co. Ltd.) were added to plant samples before grinding. After oscillated at 4 °C for 30 min, 20 ml dichloromethane were added to the crude extracts. Subsequently, the mixtures was centrifuged at 13,000 g for 5 min (4 °C), and the lower organic phase was used in ABA and GA assay. After dried by nitrogen oxides and reconstituted, the lower organic phase was injected into a liquid chromatography-tandem mass spectrometry system consisting of an poroshell 120 SB-C18 reversed-phase column (Agilent, America) and a triple quadruple tandem mass spectrometer (Quattro Premier XE; Waters). Three biological replications were performed for each test.

### Measurement of phosphorus (P) and phosphate (pi) contents in plants

Inorganic Pi measurements were performed as described previously [28]. The sample (1 mg) was homogenized with 10 μL extraction buffer (100 mM NaCl, 10 mM Tris, 1 mM EDTA, 1 mM phenylmethylsulfonyl fluoride and 1 mM β-mercaptoethanol). The homogenized sample was mixed with 1% glacial acetic with ratio of 1:9 (*V*/V) and incubated at 42 °C for 30 min. After centrifuged at 13,000 g for 5 min, 300 μL of the supernatant aliquot was added to 700 μL of assay solution (0.35% NH4MoO4, 0.86 N H2SO4, and 1.4% ascorbic acid), and was incubated at 42 °C for 30 min. Pi content was measured on a TU-1901 UV-visible spectrophotometer with absorbance at 820 nm (Beijing Purkinje General Instrument limited liability company, Beijing, China). Plant total P (TP) content was analyzed by the molybdenum blue method using H_2_SO_4_-H_2_O_2_ to digest the samples at 300 °C. Values were normalized to dry weight.

#### BCIP staining assay

Seeds were cultured on 1/2 MS medium containing 1 mM Pi. The samples were incubated in staining buffer containing 5-bromo-4-chloro-3-indolyl phosphate (BCIP) at room temperature for two hours. The commercial staining buffer (product number 0000325105) was purchased from Promega Company, USA.

## Additional files

Additional file 1: **Figure S1.** Expression profiling of *BnPHT1;4* and *BnPHT1;5* in 24-h-imbibed seeds of *Brassica napus* under Pi starvation. **Figure S2.** Expression profiling of *BnPHT1;4* in shoots and roots of *Brassica napus* under Pi starvation. **Figure S3.** Assay of total phosphorus content in seeds of the *BnPHT1;4* overexpression transgenic *Brassica napus*. **Figure S4.** Assay of thousand-grain weight of dry seeds of the *BnPHT1;4* overexpression transgenic *Brassica napus*. **Figure S5.** The effect of exogenous phosphate (Pi) on seed germination and early seedling growth of *Brassica napus*. **Figure S6.** Quantitative RT-PCR analysis of expression of *BnPAP2* and *BnPAP15* in *BnPHT1;4* overexpression transgenic *Brassica napus*. **Table S1.** Primers used in quantitative RT-PCR analysis. (PDF 464 kb)

Additional file 2: **Dataset S1.** Dynamics of the relative pool sizes (%) of the total phosphorus (P) in the *BnPHT1;4* overexpression transgenic seedlings of *Brassica napus* grown under 1 mM Pi. **Dataset S2.** Dynamics of the relative pool sizes (%) of the total phosphorus (P) in the *BnPHT1;4* overexpression transgenic seedlings of *Brassica napus* grown without Pi. **Dataset S3.** Quantitative analysis of germination rates of the *BnPHT1;4* overexpression transgenic seedlings of *Brassica napus* grown on 1/2 MS medium with 10, 50 and 100 μM abscisic acid (ABA). **Dataset S4.** Quantitative analysis of germination rates of the *BnPHT1;4* overexpression transgenic seedlings of *Brassica napus* grown on 1/2 MS medium with 100 μM gibberellins (GA) or 10 mM paclobutrazol (PAC). (XLS 51 kb)

## Abbreviations

ABA: Abscisic acid
BCIP: 5-bromo-4-chloro-3-indolyl phosphate
DAG: Days after seed germination
GA: Gibberellin
HP: P-sufficient
LP: P-deficient
P: Phosphorus
PAC: Paclobutrazol
PHT: Phosphate transporter
Pi: Phosphate
X-Gluc: 5-bromo-4-chloro-3-indolyl-β-dglucuronic acid
