# Supplementary material for: The high-affinity transporter BnPHT1;4 is involved in phosphorus acquisition and mobilization for facilitating seed germination and early seedling growth of Brassica napus
Source: BMC Plant Biol. 2019 Apr 25;19:156. doi: 10.1186/s12870-019-1765-3 (PMC6482582; doi:10.1186/s12870-019-1765-3)
Supplement: Supplementary file 1 — Figure S1. Expression profiling of BnPHT1;4 and BnPHT1;5 in 24-h-imbibed seeds of Brassica napus under Pi starvation. Figure S2. Expression profiling of BnPHT1;4 in shoots and roots of Brassica napus under Pi starvation. Figure S3. Assay of total phosphorus content in seeds of the BnPHT1;4 overexpression transgenic Brassica napus. Figure S4. Assay of thousand-grain weight of dry seeds of the BnPHT1;4 overexpression transgenic Brassica napus. Figure S5. The effect of exogenous phosphate (Pi) on seed germination and early seedling growth of Brassica napus. Figure S6. Quantitative RT-PCR analysis of expression of BnPAP2 and BnPAP15 in BnPHT1;4 overexpression transgenic Brassica napus. Table S1. Primers used in quantitative RT-PCR analysis. (PDF 464 kb) [file 12870_2019_1765_MOESM1_ESM.pdf]

Additional File 1

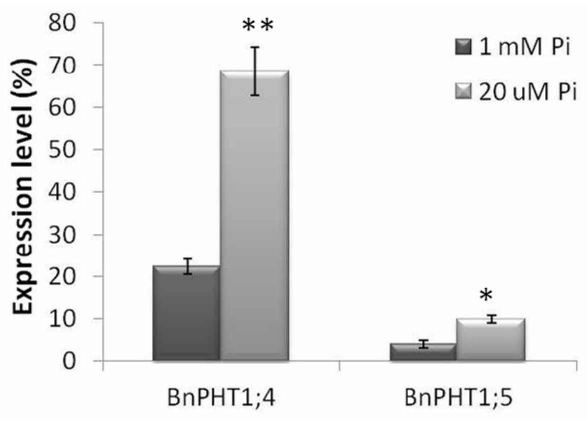

Figure S1. Expression profiling of *BnPHT1;4* and *BnPHT1;5* in 24-hours-imbibed seeds of *Brassica napus* under Pi starvation. Total RNA was isolated from 24-hours-imbibed seeds on 1/2 MS medium with 1 mM or 20 μM Pi for quantitative RT-PCR analysis, using *BnACT2* gene as standard control. The error bars indicate the standard errors. Significance of difference was analyzed by Duncan's test (P<0.05, n = 3).

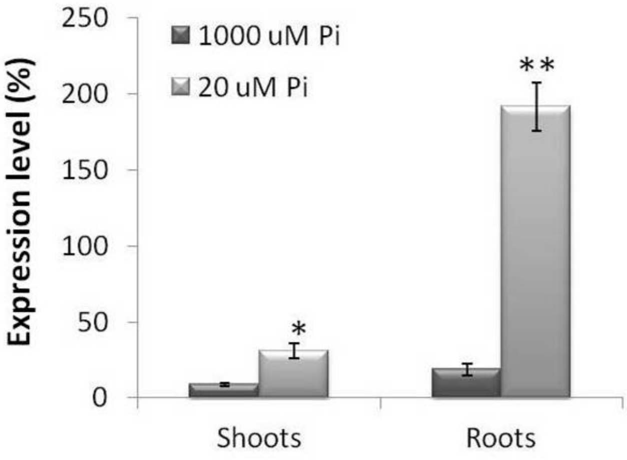

Figure S2. Expression profiling of *BnPHT1;4* in shoots and roots of *Brassica napus* under Pi starvation. The 21 DAG seedlings were transferred to 20 or 1000 μM Pi for 3 days, and then total RNA was isolated from shoots and roots for quantitative RT-PCR analysis, using *BnACT2* gene as standard control. The error bars indicate the standard errors. Significance of difference was analyzed by Duncan's test (P<0.05, n = 3).

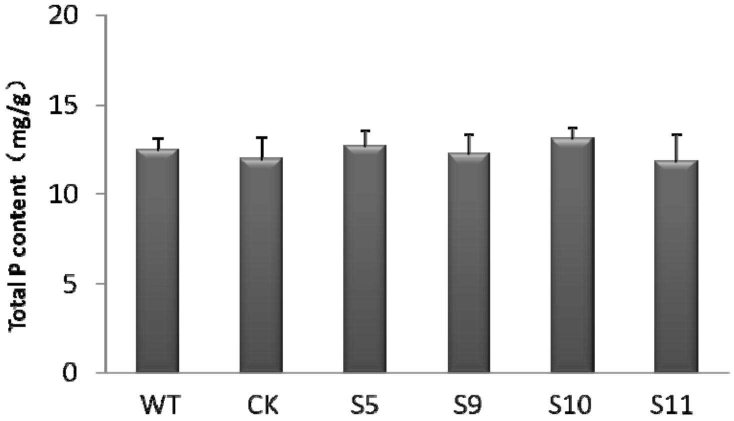

Figure S3. Assay of total phosphorus content in seeds of the *BnPHT1;4* overexpression transgenic *Brassica napus*. The error bars indicate the standard errors. Significance of difference was analyzed by Duncan's test (n=3). WT, wild type; CK, transgenic null line; S5, S9, S10 and S11, independent *BnPHT1;4* overexpression transgenic lines.

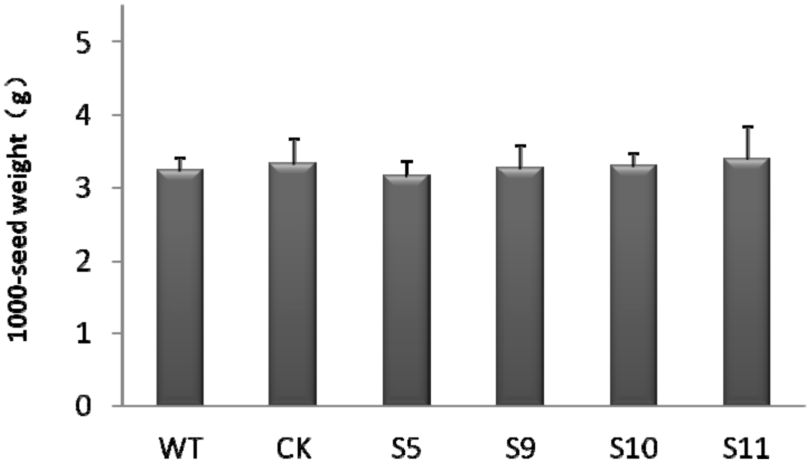

Figure S4. Assay of thousand-grain weight of dry seeds of the *BnPHT1;4* overexpression transgenic *Brassica napus*. The error bars indicate the standard errors. Significance of difference was analyzed by Duncan's test (n = 3). WT, wild type; CK, transgenic null line; S5, S9, S10 and S11, independent *BnPHT1;4* overexpression transgenic lines.

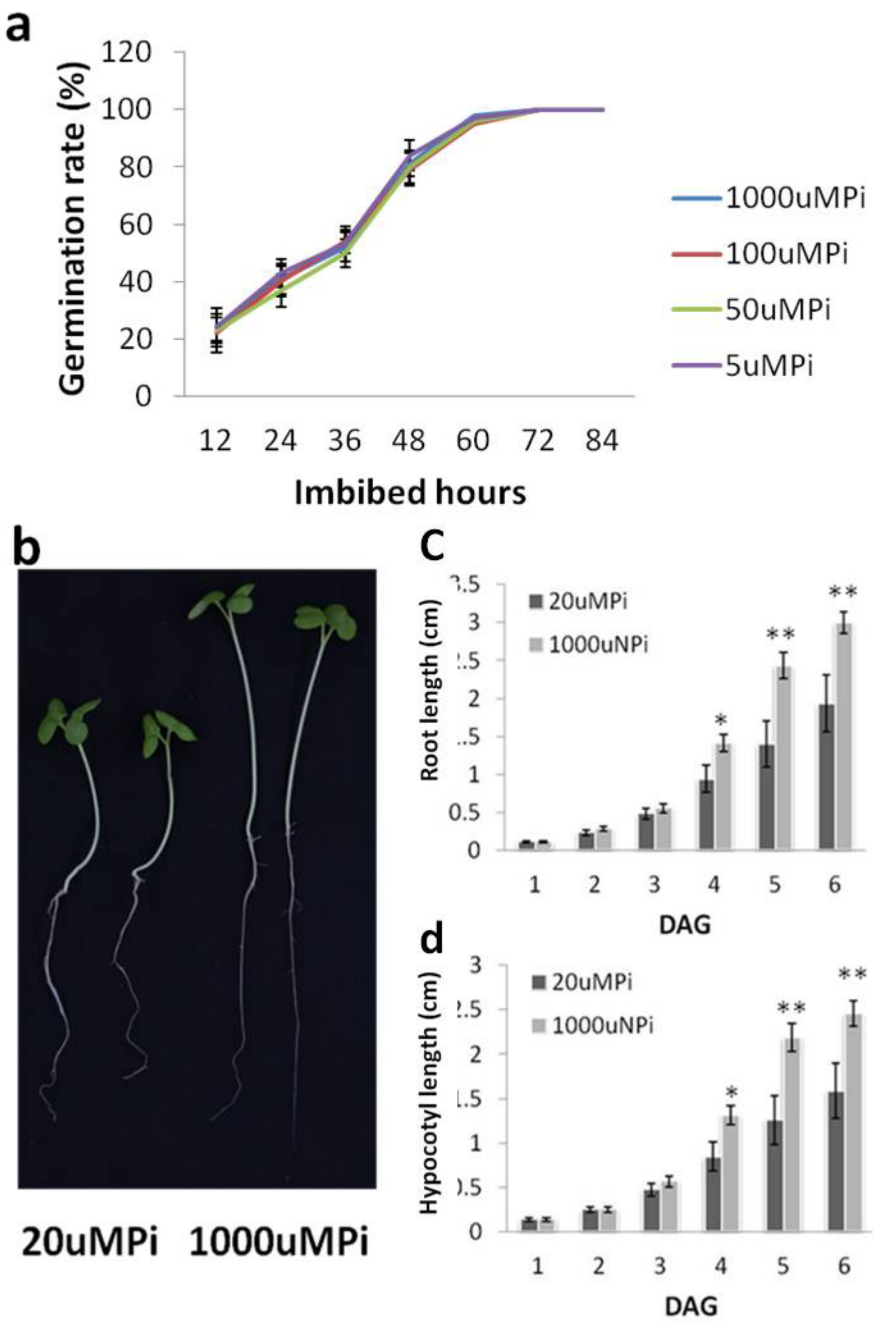

Figure S5. The effect of exogenous phosphate (Pi) on seed germination and early seedling growth of *Brassica napus*. (**a**) Germination of wild type seeds on 1/2 MS medium with different Pi concentrations. Exogenous phosphate content don't affect seed germination. The data are presented as means ± SD (n = 5). (**b**) Phenotype of the wild type seedlings grew on 1/2 MS medium containing 20 or 1000 μM Pi. Exogenous Pi affects early seedling growth. (**c** and **d**) Quantitative analysis of root length and hypocotyl length of the early growing seedlings treated with 20 or 1000 μM Pi. Significance of difference was analyzed by Duncan's test (*p＜0.05, **p＜0.01, n = 9).

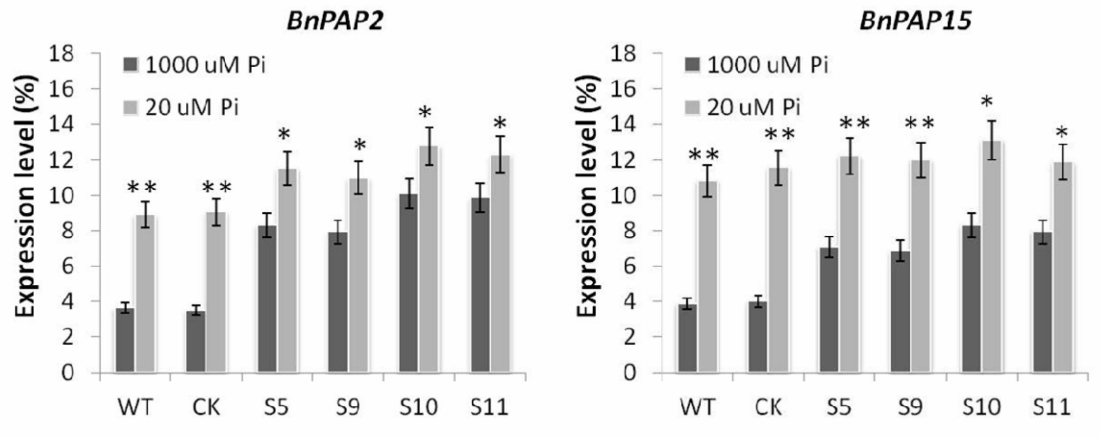

Figure S6. Quantitative RT-PCR analysis of expression of *BnPAP2* and *BnPAP15* in *BnPHT1;4* overexpression transgenic *Brassica napus*. Expression of *BnPAP2* and *BnPAP15* in 9-hours-imbibed seeds of the *BnPHT1;4* overexpression transgenic lines and controls (WT and CK) on 1/2 MS medium with 20 or 1000 μM Pi. The error bars indicate the standard errors. Significance of difference was analyzed by Duncan's test (*$p < 0.05$, **$p < 0.01$, n = 3). WT, wild type; CK, transgenic null line; S5, S9, S10 and S11, independent *BnPHT1;4* overexpression transgenic lines.

Table S1. Primers used in the real-time qRT-PCR analysis

primer names	sequence (5’-3’)
BnACT2	GACCGTATGAGCAAAGAGATCACA
	TCTCGGGAGGTGCAACGA
BnPHT1;4	GTACCGGCGGAGATCTTCCCAGC
	CTACACAATGGGGACCGTTC
BnPAP2	AGGATTAACAGTGGCCTCGT
	CGCGTGTGTTGCTTCTAGAT
BnPAP15	AGGATTAACAGTGGCCTCGT
	CGCGTGTGTTGCTTCTAGAT
BnGA1	TCCTCGCTTTGTTCGTCTCT
	GAGTGTGTCAACCTCCAACA
BnGA2	TTAAGAGAGAAAGCGCGCAA
	TTCCTTGCACTCCCTTGGAA
BnGA3	TAAGAAGCGTTGGGAGAAGC
	TCACCGTCTCTAAGCTTCCA
BnKAO	CTGAAGTCTTCCCGGATCCA
	TCCGTGTTGCTCCTTTTCAC
BnNCED5	ATGGGGCCGTTTCTTTTGTC
	TGCCAACAGAGCCTTTATTACA
BnNCED9	TTCGAGGGTTTTGGCTCAGT
	CGAAATTGGTTTGCTTTTAC
BnABI4	CCAATAGCTCACAACTCGGC
	GCTCGTTTCCTAGCAGCTTC
BnIPS1	ATGAAGAGGCAGCTGATATTCA
	ATCTGCTGATTCCGAGGGG
BnACP5	TGGAAGATCGTCGTTGGTCA
	GTCGGGTCAACATCTCCTCT
BnRNS1	GGAACACGAATGGGAGAAGC
	TGGCTATTCCCAGACCCATC
BnPHO1	TTACCGTTCCGTGACATGGA
	AAGCCAGTAGTCCCCTGTTC
BnPHT1;5	ATCCGAAGAAGACTGACGCT
	ATCTCTGTCGCATCACCTCC
GUS	ACACCGATACCATCAGCGAT
	TCTTCACTCCACATGTCGGT
